# Predicting gene expression from histone modifications with self-attention based neural networks and transfer learning

**DOI:** 10.3389/fgene.2022.1081842

**Published:** 2022-12-14

**Authors:** Yuchi Chen, Minzhu Xie, Jie Wen

**Affiliations:** College of Information Science and Engineering, Hunan Normal University, Changsha, China

**Keywords:** gene expression, histone modification, deep learning, transfer learning, convolutional neural network

## Abstract

It is well known that histone modifications play an important part in various chromatin-dependent processes such as DNA replication, repair, and transcription. Using computational models to predict gene expression based on histone modifications has been intensively studied. However, the accuracy of the proposed models still has room for improvement, especially in cross-cell lines gene expression prediction. In the work, we proposed a new model TransferChrome to predict gene expression from histone modifications based on deep learning. The model uses a densely connected convolutional network to capture the features of histone modifications data and uses self-attention layers to aggregate global features of the data. For cross-cell lines gene expression prediction, TransferChrome adopts transfer learning to improve prediction accuracy. We trained and tested our model on 56 different cell lines from the REMC database. The experimental results show that our model achieved an average Area Under the Curve (AUC) score of 84.79%. Compared to three state-of-the-art models, TransferChrome improves the prediction performance on most cell lines. The experiments of cross-cell lines gene expression prediction show that TransferChrome performs best and is an efficient model for predicting cross-cell lines gene expression.

## Introduction

Understanding the patterns of gene regulation has been one of the major focuses of biological research. A variety of biological factors are thought to be involved in the regulation of gene expression. The regulatory factors usually include transcription factors, cis-regulatory elements, and epigenetic modifications. As a type of epigenetic modifications, histone modification plays an important role in gene expression regulation (Gibney and Nolan, 2010). Nucleosome is the building block of a chromosome, which consists of an octamer of histones and 147 base pair (bp) DNA wrapping around the octamer. Since histone is a core component of the nucleosome, histone modifications directly affect the structure of chromatin and control the expression intensity of nearby genes. Recently, a great number of researches have shown that histone modifications have a great impact on gene expression, chromosome inactivation, replication, and cell differentiation (Krajewski, 2022; Lin et al., 2022). There are a variety of histone modification marks at different chromosomal locations, and there may be a set of “codes” of histone modifications to control gene expression (Peterson and Laniel, 2004). Due to the high-throughput sequencing technologies, a huge amount of histone modifications data and gene expression data are available, and using computational algorithms to predict gene expression based on histone modifications is feasible.

To date, a variety of computational methods have been used to predict gene expression based on gene regulatory factors. For example, Beer and Tavazoie, (2004) used Bayesian networks to predict gene expression from DNA sequences. Ouyang et al. (2009) used a linear regression model to predict gene expression based on 12 transcription factors. Zeng et al. (2020) combined the information of proximal promoter and distal enhancer to predict gene expression. In 2010, Karlić et al.(2010) found histone modification levels and gene expression are well correlated, and derived quantitative models to predict gene expression from histone modifications. Li et al. (2015) used a machine learning method to predict gene expression in lung cancer from multiple epigenetic data such as CpG methylation, histone H3 methylation modification and nucleotide composition. In 2016, Singh et al. (Singh et al., 2016) used a convolutional neural network (CNN) DeepChrome to predict gene expression based on five critical histone modification marks. To improve prediction accuracy, they (Singh et al., 2017) integrated attention mechanism into a neural network and proposed a prediction model AttentiveChrome. Temporal Convolutional Network (Zhu et al., 2018; Kamal et al., 2020) is also utilized to predict the gene expression from histone modifications. In 2022, Hamdy et al. (2022) proposed three variations of CNN models called ConvChrome.

Though the above methods have achieved good performances, there are still room for improvement, and some recently emerging technologies have provided some ways. When models are trained and tested on different cell lines which is knowns as cross-cell lines prediction, the model performance is always compromised. For example, compared to training and testing on the same cell line dataset, the average prediction accuracy of DeepChrome trained on other cell lines is 2.3% lower. Because of the large variety of cell lines, it is difficult to obtain histone modification data and gene expression data for all types of cell lines. Therefore predicting gene expression using models trained on other cell lines is useful and in urgent need.

Transfer learning is a machine learning technique in which a model trained on a specific task is reused as part of the training process for another similar task (Tan et al., 2018). Transfer learning allows training and prediction using the dataset from different sources with similar characteristics and significantly reduces dataset bias. Transfer learning has achieved great success in prediction tasks that require learning transfer features (Sun et al., 2022; Zhu et al., 2022). In the field of bioinformatics, transfer learning enables existing trained models to efficiently work on similar datasets that are lack of labels, which reduces the cost of biological experiments.

In the paper, we propose a neural network model TransferChrome with self-attention mechanism and transfer learning to predict gene expression based on histone modifications data. TransferChrome uses neural network layers with self-attention mechanism to capture global contextual information of data. In order to correct the data bias of cross-cell lines gene expression prediction, we used transfer learning. The experimental results show that TransferChrome achieved an average Area Under the Curve (AUC) score of 84.79%, which is better than other 3 state-of-the-art similar models. The cross-cell lines prediction experiments also show that TransferChrome outperforms other models.

## Materials and method

### Data collection and processing

The experimental data comes from the Roadmap Epigenome Project (REMC) (Kundaje et al., 2015), which consists of 56 cell lines’ histone modifications data and the corresponding normalized RPKM expression data of 17170 samples. Same as DeepChrome (Singh et al., 2016), five histone modification marks that play important roles in gene expression were selected for our experiments. These 5 marks include H3K4me3, H3K4me1, H3K36me3, H3K27me3, and H3K9me3. Their functional categories are summarized in Table 1. Each sample in the dataset represents a gene. The data of one sample include the five histone modification marks signal within 10000bp upstream and downstream of the transcription start sites (TSSs) of the corresponding gene.

**TABLE 1:** Five core histone modification marks and their functional categories.

Histone mark	Associated regions	Functional category
H3K27me3	Polycomb repression	Repressor mark
H3K36me3	Transcribed regions	Structural mark
H3K4me1	Enhancer regions	Distal mark
H3K4me3	Promoter regions	Promoter mark
H3K9me3	Heterochromatin regions	Repressor mark

**FIGURE 1:**
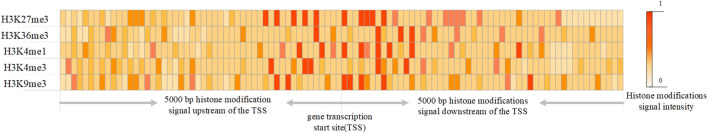
The data structure representing histone modifications.

According to DeepChrome (Singh et al., 2016), the 10000 bp is equally divided into 100 bins and the histone modifications data of one sample is encoded into an *n* × *m* matrix *x*, where *n* is the number of histone modification marks and *m* is the number of bins (see Figure 1). The histone sequencing data provided by REMC were quantified by BEDTools into histone modification signals. Therefore, *x*_*i*,*j*_ represents the signal of the *j*-th histone modification mark in the *i*-th bin.

Since the normalization of training data can speed up the convergence of model training and allows the model to fit the data better (Singh and Singh, 2020), the z-score method is used to normalize the data for each histone modification mark as Eq. 1. In Eq. 1, ${\hat{x}}_{i,h}$ represents the normalized signal of *h*-th histone modification mark in the *i*-th bin. ${\bar{x}}_{h}$ and *σ*_*h*_ denote the mean and standard deviation of the signals of the *h*-th histone modification mark of all genes in a cell line.

$${\hat{x}}_{i,h}=\frac{x_{i,h}-{\bar{x}}_{h}}{\sigma_{h}}$$ (1)

According to previous studies (Singh et al., 2016), each gene is assigned a label based on its expression value. The median of expression values of all genes in a given cell line is denoted as *t*. If the expression value of a gene is higher than or equal to *t*, it is labeled with 1; otherwise it is labeled with 0.

To avoid interfering from adjacent genes, those genes whose TSSs are within 5000 bp downstream of previous kept genes’ TSSs are deleted. At last there are 17170 genes remained.

### Design of neural network model

**FIGURE 2:**
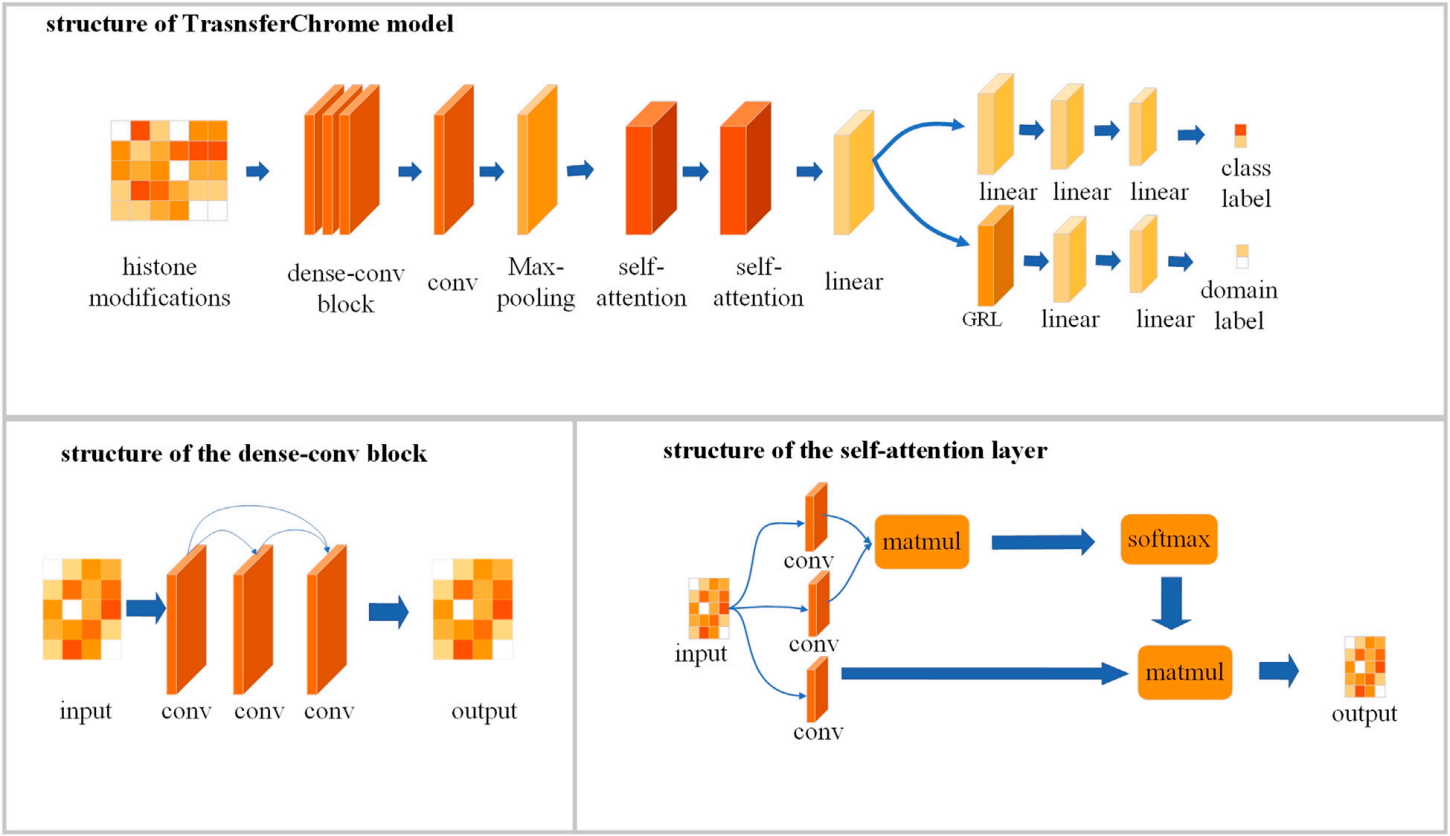
The model structure of TransferChrome.

As shown in Figure 2, TransferChrome is composed of multiple modules: a feature extraction module, a label classification module and a domain classification module. The feature extraction module is used to calculate the latent features of data. It includes a dense-conv block, a 1D convolutional layer, a 1D max pooling layer, two self-attention layers, and a linear layer (also called fully connected layer or dense layer). The label classification module predicts a gene expression label. It includes three linear layers. The domain classification module predicts a domain label, which includes a gradient reversal layer (GRL) and two linear layers. It learns transfer features, which allows the model to achieve better performance in cross-cell lines gene expression prediction.

### Dense-connected convolutional layer for extracting local features of data

To make the model better capture the features of the data, we optimized the convolutional neural network in the feature extraction module. The convolutional neural network uses feature detectors, also known as convolution kernals or filters to capture data’s features. According to its size, a convolution kernal will aggregate all the information in the receptive field to extract a corresponding feature. By increasing the number of convolutional layers, a model can learn more complex features. However, the deepened network structure easily ignores the features captured by earlier convolutional layers, which usually represent the simple but also basic features of the data. Densely Connected Convolutional Networks (DenseNet) (Huang et al., 2017) uses a method called dense connectivity pattern enhances the reusability of features. Compared with the classic Convolutional Network, DenseNet connects convolutional layers densely so that the feature extracted by each layer could be used repeatedly. DenseNet performs a deep supervision and strengthen the weights of features captured by earlier convolutional layers. Inspired by DenseNet, a dense-conv block that contains several densely connected convolutional layers is used to extract features in our model. A dense-connected convolutional layer is a convolutional layer which connected to all other convolutional layers directly. It means that the input of a dense connected convolutional layer not only comes from its adjacent convolutional layer, but also from other preceding layers. The dense-conv block allows the model to learn the complex features of the data while also ensuring that the low-level convolutional layer retains a greater influence in the model’s decision-making.

Let *x*_*l*_ be the output of the *l*th densely connected convolutional layer. The input of the *l*th dense connected convolutional layer contains the outputs of all the previous *l*−1 layers as Eq. 2 shows.

$$x_{l}=H\left(\left[x_{0},x_{1},\ldots \ldots x_{l-1}\right]\right)$$ (2)

[*x*_0_, *x*_1_, ……*x*_*l*−1_] refers to the concatenation of the feature-maps output from preceding layers. The composite function *H* concludes a rectified linear unit (RELU) and 1D convolutional layer.

In TransferChrome, the dense-conv block consists of three dense-connected convolutional layers (kernal number = 32, 16, 8 and kernal length = 5, 5, 5). The dense-conv block is followed by a convolutional layer (kernal number = 50 and kernal length = 5) and a max-pooling layer (kernal length = 2). A dropout layer is added after each convolutional layer and the dropout rate is 0.4. The output of the max-pooling layer is input into a following self-attention layer.

### Self-attention layer for aggregating global information

Regulatory factors at different locations may interact and act on gene expression together. Therefore effective integration of upstream and downstream information in the genome usually leads to better computational results (Ji et al., 2021). The Transformer (Vaswani et al., 2017) is an efficient neural network model which has achieved good results in many fields such as natural language processing (Devlin et al., 2019) and image recognition (Dosovitskiy et al., 2021). The self-attention mechanism used in Transformer can effectively integrate data’s global features. The self-attention mechanism also has been widely adopted in the field of Bioinformatics (Avsec et al., 2021; Ji et al., 2021). For example, the researchers used this mechanism to significantly improve the regulatory elements prediction from genomic DNA sequences (Avsec et al., 2021). Previous experiments (Singh et al., 2017, 2016) have illustrated that histone modifications closer to gene’s TSS have greater influence in the gene expression. We add self-attention mechanism to the model, and use a position encoding function to concatenate input data with relative distance information. The relative distance information contains the relative distance between each point and the TSS point, and the output of the position encoding function is denoted by *x*.

TransferChrome contains two self-attention layers to capture the long-distance dependence. The function of each self-attention layer is as Eqs 3–6.

$$Q=conv_{q}\left(x\right)$$ (3)

$$K=conv_{k}\left(x\right)$$ (4)

$$V=conv_{v}\left(x\right)$$ (5)

$$Attention\left(Q,K,V\right)=soft\max\left(QK^{T}\right)V$$ (6)

Each self-attention layer uses three one-dimensional convolutional layers (kernal length = 1) *conv*_*q*_, *conv*_*k*_, and *conv*_*v*_ to calculate a query matrix *Q*, a key matrix *K* and a latent variable matrix *V*, respectively. The number of output channels of *conv*_*q*_ and *conv*_*k*_ is half of the number of input channels, and the number of output channels of *conv*_*v*_ is equal to the number of input channels. Then the self-attention layer calculate data’s attention score matrix *QK* ^*T*^ by multiplying (matmul) *Q* and *K*. Finally, the attention score matrix will be normalized by a softmax function, and multiplied with *V*. At the end of the feature extraction module, there is a linear layer following the last self-attention layer. The feature extraction module outputs a low-dimensional feature vector. Then the feature vector is inputted into the label classification module and the domain classification module at the same time.

### Label classification module and domain classification module

The label classification module predicts the gene expression label of the sample, which is the main task of our model. It is a binary classification task with 0 and 1 represent low expression and high expression, respectively. According to Long et al. (2015) and Ganin and Lempitsky (2015), domain adaption can improve prediction accuracy in transfer learning. Therefore, TransferChrome uses a domain classification module for cross-cell lines prediction. It contains a GRL and two linear layers. GRL acts as an identity transform in the forward propagation of the model. In the backward propagation, GRL takes the gradient from the subsequent layer and multiplies it by a parameter −*λ* with *λ* > 0 and passes it to the preceding layer.

The domain classification module predicts whether the sample belongs to the target domain or the source domain. Source domains are the cell lines whose genes have gene expression labels, and the cell lines whose gene have no known gene expression information are called target domains. It is also a binary classification task with 0 and 1, where 0 indicates that the sample is from the target domain and 1 indicates that the sample is from the source domain. In cross-cell lines prediction, we try to extract those features that can not be used to discern the data domain.

### Model training

For model training, we chose cross entropy as the loss function:

$$L=-\left[y\log\hat{y}+\left(1-y\right)\log\left(1-\hat{y}\right)\right].$$ (7)

Let *G*_*f*_ and *θ*_*f*_ be the function and the parameters of the feature extraction module, respectively. Let *G*_*d*_ (*G*_*y*_) and *θ*_*d*_ (*θ*_*y*_) be the function and the parameters of the domain (label) classification module, respectively. For the single cell line gene expression prediction task, the optimization goal of model training is to minimize the loss *L*_*y*_ of the label classification module without considering the domain classification module.

For the cross-cell lines gene expression prediction task, we train TransferChrome using the complete dataset from a source domain and a part of the dataset from a target domain to capture transfer features in different cell lines, and aim to minimize the objective function in Eq. 8.

$$\begin{array}{c} E\left(\theta_{f},\theta_{y},\theta_{d}\right)\\ \begin{array}{c} =\overset{N}{\underset{i=1}{\sum }}L_{\text{y}}\left(G_{y}\left(G_{f}\left(x_{i};\theta_{f}\right);\theta_{y}\right),y_{i}\right)\\ -\lambda \overset{N}{\underset{i=1}{\sum }}L_{d}\left(G_{d}\left(G_{f}\left(x_{i};\theta_{f}\right);\theta_{d}\right),d_{i}\right) \end{array}\\ =\overset{N}{\underset{i=1}{\sum }}L_{y}^{i}\left(\theta_{f},\theta_{y}\right)-\lambda \overset{N}{\underset{i=1}{\sum }}L_{d}^{i}\left(\theta_{f},\theta_{d}\right), \end{array}\;$$ (8)

where *L*_*d*_ is the loss function of the domain classification module.

In the training process, stochastic gradient descent (SGD) is used to update *θ*_*y*_ and *θ*_*d*_ to minimize the label classification loss *L*_*y*_ and *L*_*d*_. In the backward propagation, the first layer GRL of the domain classification module reverses the gradient by multiplying a negative number −*λ* and backward propagates it to the feature extraction module. After the model training, *G*_*f*_ is expected to extract transfer features in different cell lines. In the training process, the learning rate is set to 0.001, momentum is 0.85, and weight decay is 0.001. We set the max training epochs to 200 and adopted early stop strategy.

In the following single-cell line prediction experiments, each cell line data was partitioned into a training set, a validation set and a test set as DeepChrome (Singh et al., 2016). For cross-cell lines prediction, we used the source domain data and half of the target domain data to train our model, and used the other half of the target domain data as the test set.

## Experiments

### Comparison with other existing state-of-the-art methods

**FIGURE 3:**
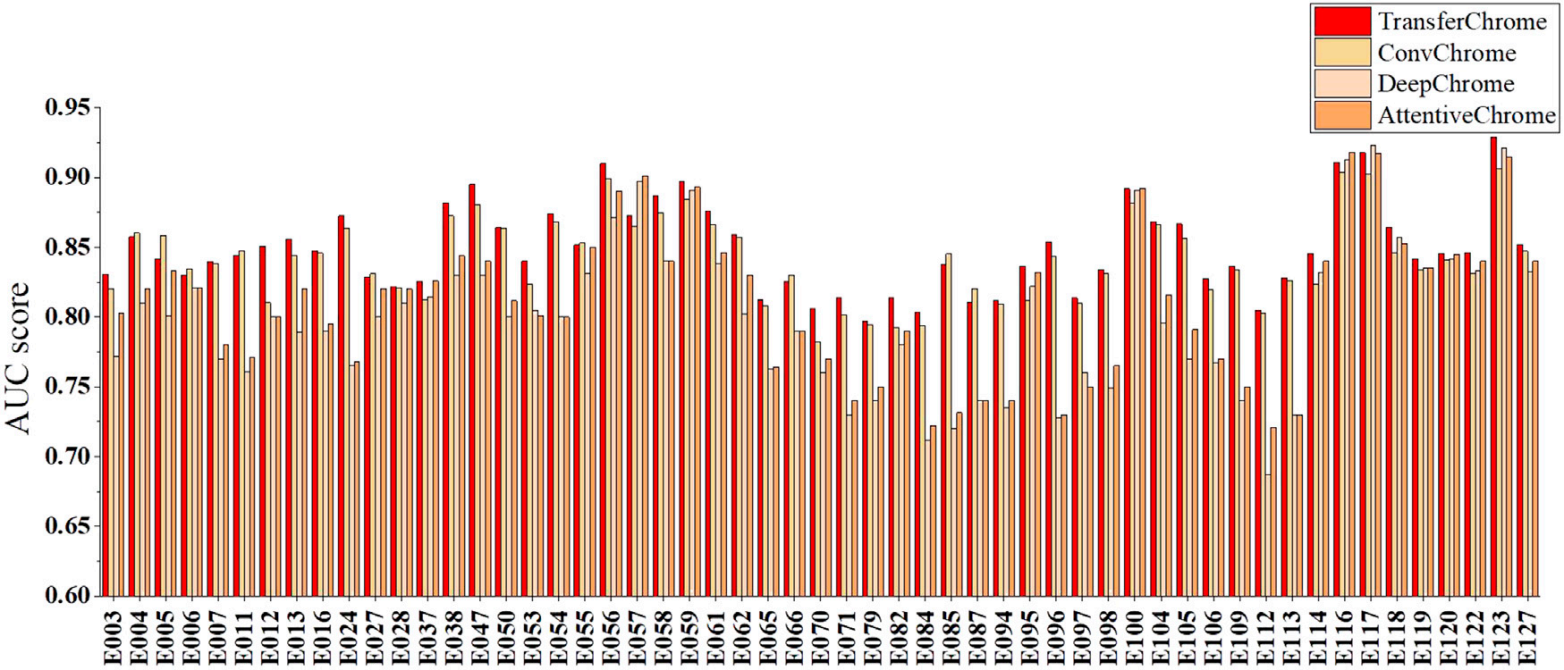
Single cell line gene expression prediction performance comparison on 56 cell lines of the models.

To evaluate the effectiveness of TransferChrome, we compared it with three state-of-the-art models (DeepChrome, AttentiveChrome, and ConvChrome_CNN1D). DeepChrome (Singh et al., 2016) is a convolutional neural network. It consists of a convolutional layer (convolution kernal size is 10, the number of convolution kernals is 50), a max pooling layer (convolution kernal size is 10), and two fully connected layers (the number of units is 900, 125). AttentiveChrome (Singh et al., 2017) is a recurrent neural network that uses two attention mechanisms. ConvChrome (Hamdy et al., 2022) includes three variations of CNN models, among which ConvChrome_CNN1D achieved the best performance. In the experiments, all models used the same five types of core histone modifications from the REMC project to predict gene expression. In the experiments of single-cell gene expression prediction, we did not use the domain classification module of TransferChrome and only used the label classification module. DeepChrome was implemented and trained according to Singh’s paper (Singh et al., 2016). For AttentiveChrome, we used the trained model downloaded from http://kipoi.org/models/AttentiveChrome/. Because ConvChrome’s code and data are not available, We implement ConvChrome_CNN1D with PyTorch. We used the AUC score as our evaluation metric. Experimental results on 56 cell lines are shown in Figure 3 and Table 2, compared with the other models, TransferChrome improved the prediction accuracy on most cell lines. TransferChrome has a significant improvement in average AUC compared to DeepChrome and AttentiveChrome. Compared with ConvChrome, TransferChrome also has better performance.

**TABLE 2:** The minimum, mean, maximum, and median of the AUC scores of single cell line gene expression prediction of the models on 56 cell lines.

	Min	Mean	Max	Median
TransferChrome	0.7972	0.8479	0.9289	0.8449
ConvChrome	0.7820	0.8399	0.9061	0.8386
DeepChrome	0.6871	0.8003	0.9236	0.8019
AttentiveChrome	0.7221	0.8093	0.9197	0.8216

### Cross-cell lines gene expression prediction performance comparison

**FIGURE 4:**
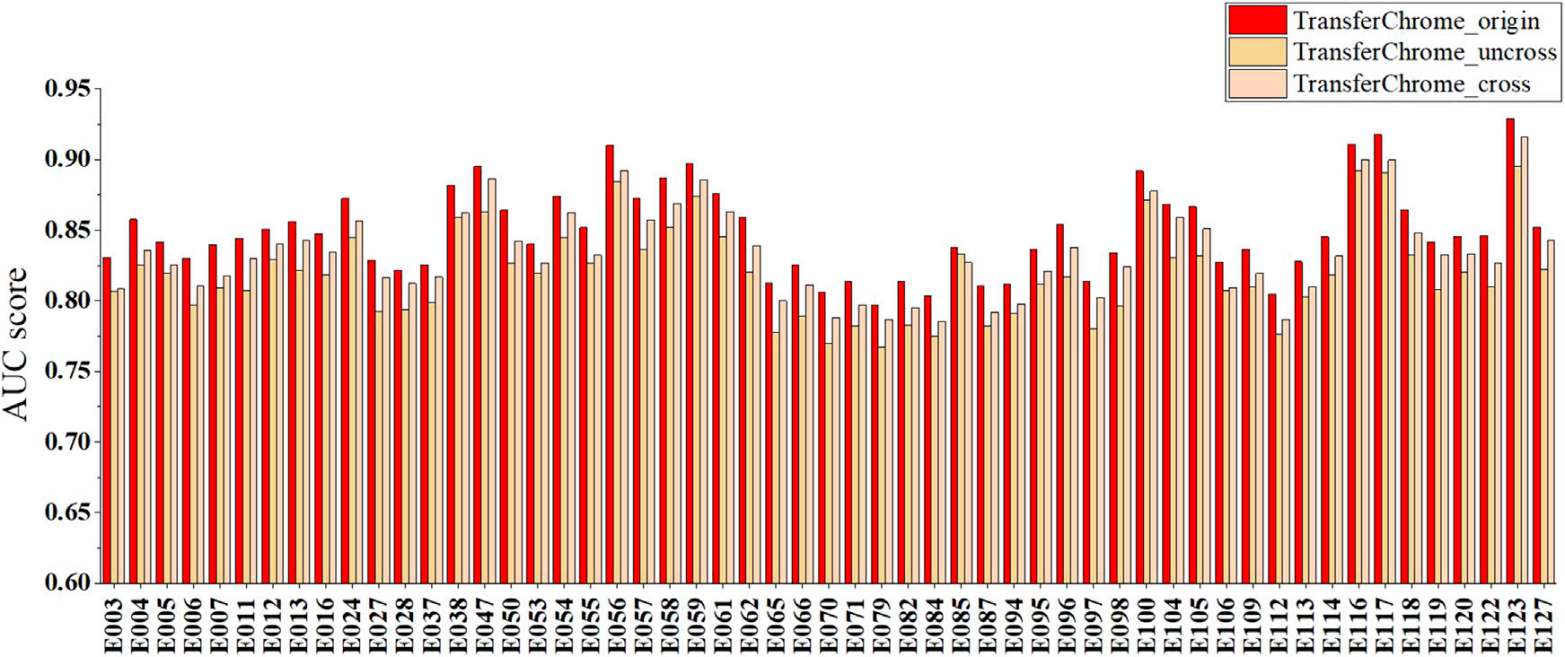
The performance comparison of different versions of TransferChrome: TransferChrome_cross, TransferChrome_uncross and TransferChrome_origin.

For the performance comparison in cross-cell lines gene expression prediction, we arbitrarily selected a cell line (E085) as the source domain and each one of other cell lines as the target domain. Figure 4 and Table 3 show the experimental results. In Figure 4 and Table 3, TransferChrome_cross means that TransferChrome was trained and tested on different cell lines. TransferChrome_uncross and TransferChrome_origin did not use the domain classification module, and TransferChrome_origin was trained and tested on the same cell lines, while TransferChrome_uncross was trained and tested on different cell lines. Table 3 shows the performance comparison of TransferChrome, DeepChrome and ConvChrome in cross-cell lines gene expression prediction. In Table 3, DeepChrome and ConvChrome indicate that the models were trained and tested on the same cell line, while DeepChrome_uncross and ConvChrome_uncross indicate that those models were trained with E085 cell line’s data but were tested on other cell lines.

**TABLE 3:** Comparison of the minimum, mean, maximum, and median of the AUC scores of TransferChrome, DeepChrome and ConvChrome in single cell line and cross-cell lines gene expression predictions.

	Min	Mean	Max	Median
TransferChrome_origin	0.7972	0.8479	0.9289	0.8449
TransferChrome_uncross	0.7672	0.8185	0.8950	0.8182
TransferChrome_cross	0.7866	0.8330	0.9156	0.8300
DeepChrome	0.6871	0.8003	0.9236	0.8019
DeepChrome_uncross	0.6652	0.7737	0.8951	0.7710
ConvChrome	0.7820	0.8399	0.9061	0.8386
ConvChrome_uncross	0.7571	0.8111	0.8791	0.8076

The results have shown that the average AUC of TransferChrome_uncross trained in a E085 cell line and tested on another dropped by 2.9% compared to those of TransferChrome_origin trained and tested on a same cell line. Similarly, the average AUCs of DeepChrome_uncross and ConvChrome_uncross dropped by 2.6% and 2.9% compared to those of DeepChrome and ConvChrome, respectively. Though TransferChrome_cross did not achieve the same effect as TransferChrome_origin, the average AUC drop is reduced to 1.5%, which showed that using domain classification module indeed improves the performance in cross-cell lines prediction.

### Contributions of dense connectivity pattern and different position encoding functions

We carried experiments to see whether dense connectivity pattern and different position encoding functions have obvious impact on the performance of TransferChrome. A total of 9 cell lines out of 56 with worst (E079, E084, E112), median (E114, E120, E128), and best (E116, E117, E123) AUC scores were selected for ablation experiments.

**FIGURE 5:**
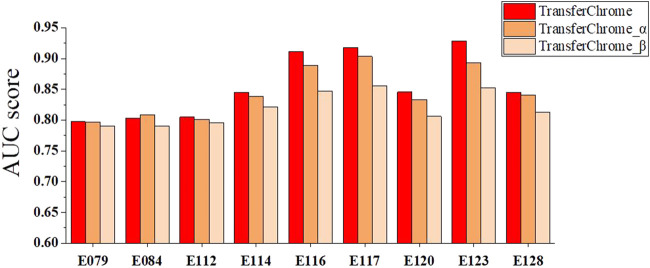
The performance comparison of different versions of TransferChrome: TransferChrome, TransferChrome_*α* and TransferChrome_*β* on 9 cell lines (E079, E084, E112, E114, E120, E128, E116, E117, E123).

We compared three TransferChrome model variations to discuss the contribution of different position encoding functions. The position encoding function adopted by TransferChrome calculates the relative distance between TSS and bins. TransferChrome_*α* use sinusoidal position encoding (Vaswani et al., 2017) as the position function. Sinusoidal position encoding function calculates position information with a mix of sine and cosine functions. TransferChrome_*β* is the TransferChrome without adding position information. The experimental results are shown in Figure 5.

Histone modifications at different positions have different importance for gene expression prediction. Since TransferChrome_*β* ignores sequence position information, TransferChrome_*β* performance worser than TransferChrome and TransferChrome_*α*. Meanwhile, histone modifications which are close to TSS might have more significant effect on gene expression (Cheng et al., 2011). Accordingly, bins near to TSS should be assigned with higher weights for gene expression prediction (Singh et al., 2016). TransferChrome makes good use of relative distances between bins and TSS and performs better than TransferChrome_*α*.

**FIGURE 6:**
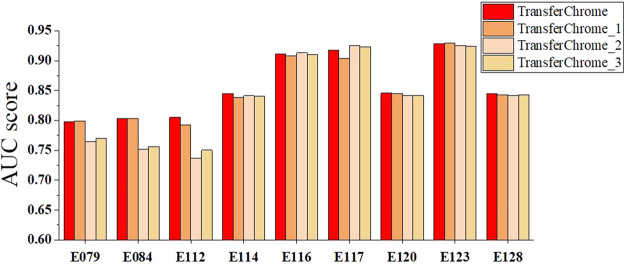
The performance comparison of different versions of TransferChrome: TransferChrome, TransferChrome_1, TransferChrome_2 and TransferChrome_3 on 9 cell lines (E079, E084, E112, E114, E120, E128, E116, E117, E123).

We also conducted comparative experiments to discuss the contribution of the dense connectivity pattern and convolutional layer kernal numbers. As shown in Figure 6, four TransferChrome model variations are compared. TransferChrome has a dense-conv block, which has three dense-connected convolutional layers with different kernal numbers (32, 16, 8). TransferChrome_1 changes the structure of the dense-conv block. Dense-conv block of TransferChrome_1 uses three dense connected convolutional layers with 50 kernals. TransferChrome_2 and TransferChrome_3 do not use dense-conv block. TransferChrome_2 only has a convolutional layer with 50 kernals. TransferChrome_3 uses three convolutional layers with 50 kernals. Figure 6 shows the experimental results of above models. On 3 cell lines E079, E084 and E112, Transferchrome and TransferChrome_1 perform significantly better than others. TransferChrome uses fewer kernals than TransferChrome_1 but achieves a similar performance.

## Conclusion and discussion

We proposed a new model called TransferChrome to predict gene expression levels based on histone modifications. TransferChrome uses self-attention mechanism to capture the long-distance dependence, and to learn hidden information features from the histone modifications data. Furthermore, TransferChrome adopts dense connectivity pattern to improve the feature exaction ability of convolutional neural network. Experimental results on the benchmark dataset of 56 cell lines showed that TransferChrome performed better than other 3 similar state-of-the-art models. To improve cross-cell lines gene expression prediction performance, TransferChrome uses transfer learning. Transfer learning makes the model capable of learning common features among different cell lines and reduces the data biases of different cell lines. Our experiments demonstrated that TransferChrome achieved the best accuracy in cross-cell lines gene expression prediction. We believe that it is useful to use transfer learning to improve cross-cell lines prediction accuracy. So far, gene expression prediction methods from histone modification data are mostly based on the five core histone modification marks. In future work, we will use more information from the histone modification data to predict gene expression. We also intend to increase the interpretability of the model in order to analyze the contribution of different histone modification marks on gene expression prediction.

## Data Availability

This study processed and analyzed publicly available data sets. These data can be found here: https://egg2.wustl.edu/roadmap/webportal/index.html.
